# Supplementary figures and images for: Selective neuroimmune modulation by type I interferon drives neuropathology and neurologic dysfunction following traumatic brain injury
Source: Acta Neuropathol Commun. 2023 Aug 18;11:134. doi: 10.1186/s40478-023-01635-5 (PMC10436463; doi:10.1186/s40478-023-01635-5)

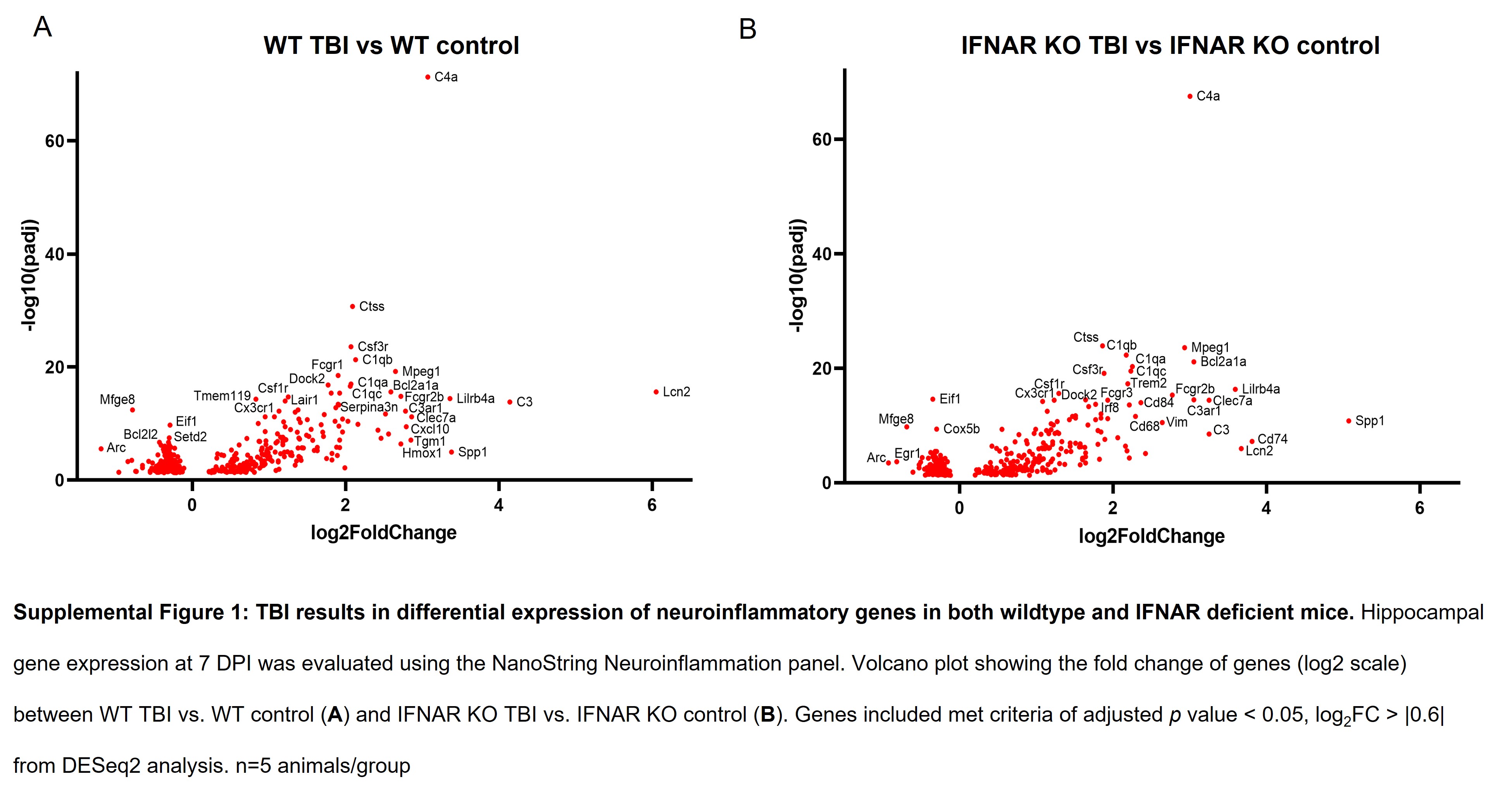

Supplement: Supplementary file 3 — Supplementary Material 3: TBI results in differential expression of neuroinflammatory genes in both wildtype and IFNAR deficient mice [file 40478_2023_1635_MOESM3_ESM.jpg]
